# Multidisciplinary treatment and immunotherapy improved the prognosis of advanced small intestine sarcomatoid carcinoma

**DOI:** 10.1093/pcmedi/pbae014

**Published:** 2024-07-02

**Authors:** Bo Wang, Meng Qiu

**Affiliations:** Colorectal Cancer Center, West China Hospital, Sichuan University, Chengdu 610041, China; Colorectal Cancer Center, West China Hospital, Sichuan University, Chengdu 610041, China

Dear Editor,

Small intestine sarcomatoid carcinoma (SISCA) is a rare type of malignancy, with an annual incidence rate of 0.5–0.8 cases per 100 000 people [1,2]. It is characterized by the presence of both cancerous and sarcomatous components [3]. Currently, there is no standardized treatment for SISCA. In cases of advanced disease, patients typically undergo chemotherapy regimens similar to those used for colorectal tumors, such as fluorouracil, oxaliplatin, and irinotecan. There have been no reported cases of targeted therapy or immunotherapy for SISCA. Here, we present a case of a male patient with tumor mutational burden-high (TMB-H) SISCA with liver and lung metastases that showed a favorable response to multidisciplinary treatment. It may provide valuable information for future treatment.

**Figure 1.**
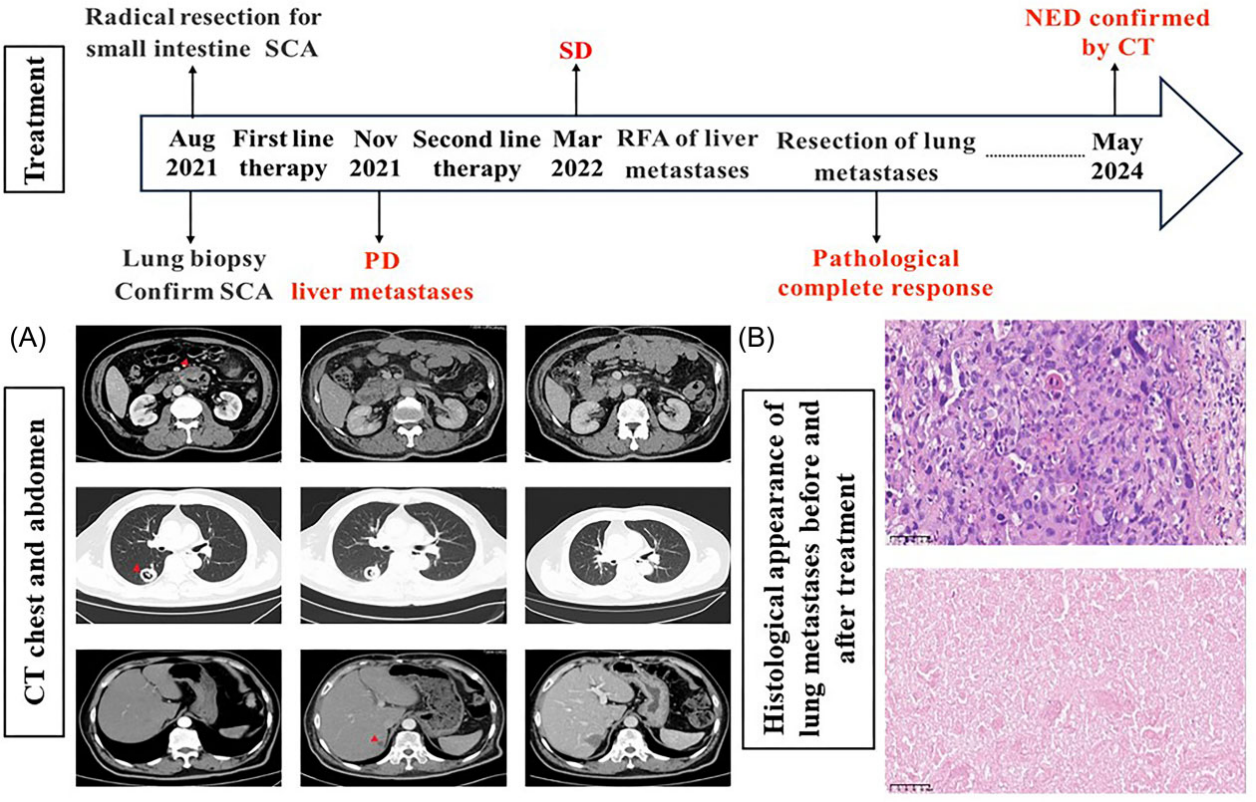
Treatment details, imaging radiographic findings and lung metastases histological appearance. (**A**) Images of chest and abdomen CT at different time points. (**B**) Histological appearance of lung metastases before and after treatment (×400 magnification). PD, Progressive disease; SD, stable disease.

In July 2021, a 61-year-old male presented with a 1-month history of abdominal pain. Computed tomography (CT) scans revealed a mass at the junction of the duodenum and jejunum, and a thick-walled cavity with solid nodules in the posterior segment of the right lower lung lobe (Fig. 1A). Gastrointestinal endoscopy identified an ulcerative neoplasm in the horizontal section of the duodenum, with preliminary pathology suggesting malignancy. Due to the potential for further growth leading to intestinal obstruction, radical surgical resection of the small intestine tumor was performed in August 2021. Postoperative pathology indicated sarcomatoid carcinoma (SCA) infiltrating the entire intestinal wall, involving nerve tissue, with tumor thrombus in blood vessels. Examination of one superior mesenteric vein, one artery node and five peri-intestinal lymph nodes revealed no tumor metastasis (pT3N0Mx). Immunohistochemistry showed programmed cell death ligand 1 (PD-L1) had a tumor proportion score of 35% and a combined positive score of 45. Next-generation sequencing of the small intestine specimen revealed TMB-H (13Mut/Mb). Then a lung lesion biopsy confirmed SCA. Following multi-disciplinary discussion, we considered that postoperative chemotherapy was necessary and that the patient would benefit more from combination immunotherapy because of the high PD-L1 expression and TMB-H of tumor cells. So the patient adopted Nab-paclitaxel (100 mg/m² day 1, every 2 weeks), oxaliplatin (85 mg/m² day 1, every 2 weeks), leucovorin (400 mg/m² day 1, every 2 weeks), and fluorouracil (1200 mg/m² day 1–day 2, every 2 weeks) combined with pembrolizumab (200 mg day 1, every 3 weeks) for three cycles. A reassessment revealed disease progression with new liver metastases (Fig. 1A). The treatment plan was changed to irinotecan (190 mg/m² day 1, every 3 weeks) and capecitabine (800 mg/m² twice a day, day 1–day 14, every 3 weeks) combined with bevacizumab (7.5 mg/kg, day 1, every 3 weeks). Unfortunately, the patient experienced severe febrile neutropenia (FN), leading to the discontinuation of anti-tumor treatment. After recovery from FN, the patient underwent another two cycles of reduced-dose treatment. Five months after receiving postoperative therapy, a follow up revealed no significant changes in lung and liver lesions. After multidisciplinary discussion again, we believed that systemic treatment plus local treatment of liver and lung lesions may achieve better results than systemic treatment alone. So radiofrequency ablation (RFA) was performed on the liver lesion, and surgical removal was conducted for the lung lesion, with no residual tumor observed in the postoperative pathology examination of the lung (Fig. 1B). The patient has since entered the follow-up stage, and the latest routine checkup in May 2024 indicated no evidence of disease (NED).

SCA is documented in the literature to occur in various locations such as the respiratory tract, gallbladder, esophagus, stomach, kidney, and bladder. Primary SCA originating in the small intestine is extremely rare, with fewer than 55 reported cases to date. Clinical manifestations typically include symptoms of intestinal obstruction or intussusception. SISCA primarily affects middle-aged and elderly men [4]. The diagnosis of SISCA is primarily based on histological appearance and immunohistochemical evidence. This type of tumor is less sensitive to radiotherapy and chemotherapy. In a study summarizing 20 patients by Reid-Nicholson *et al*. [4], 14 patients died within 2 months to 3 years after diagnosis. Additionally, the tumor exhibits low differentiation, strong invasiveness, high propensity for metastasis, and poor prognosis, with a 5-year survival rate close to 0% [5]. The initial treatment modality for early-stage patients is surgical intervention; for advanced-stage patients, chemotherapy is employed as the primary treatment approach, with reference to colorectal cancer protocols. In recent years, with the rise of immunotherapy, promising results have been achieved in the treatment of microsatellite instability high or TMB-H colorectal cancer. Studies such as KEYNOTE-158, TAPUR, and MyPathway suggest that TMB-H and high PD-L1 expression are potential therapeutic targets in gastrointestinal tumors, improving patient progression-free survival and overall survival [6–8]. However, there have been no reports on the use of immunotherapy in SISCA.

We adopted a staged approach involving chemotherapy combined with immunotherapy, chemotherapy combined with targeted therapy, and local treatments. To date, the patient has shown no recurrence or metastasis, and disease-free survival has reached 24 months. Previous literature suggests that patients with small intestinal SCA typically succumb within 3 years; this patient has been diagnosed for nearly 3 years without recurrence or metastasis, indicating several factors contributing to a favorable prognosis. (i) The patient's TMB-H and high PD-L1 expression result in greater benefits from immunotherapy. Although new liver lesions appeared after three cycles of immunotherapy, they are likely pseudo-progression [9]. (ii) The stable and localized liver and lung lesions prompted the multidisciplinary team to opt for aggressive local treatment.

It is important to note that the patient experienced drug-related adverse reactions during treatment, which underscores the need for close monitoring of toxic side-effects during treatment and individualized treatment adjustments. However, due to the rarity and high heterogeneity of the disease, more cases and clinical research are needed to further clarify the optimal treatment options and prognostic factors.

In conclusion, the multi-disciplinary treatment provided clinical benefits for the reported case of SISCA. Molecular profiling provided a precise, individualized guide. We believe that SISCA patients with markers of benefiting from immunotherapy should be actively treated with immunotherapy and the appropriate time for local treatment sought to improve their prognosis.
